# Supplementary material for: Estimating Epidemic Incidence and Prevalence from Genomic Data
Source: Mol Biol Evol. 2019 May 6;36(8):1804–16. doi: 10.1093/molbev/msz106 (PMC6681632; doi:10.1093/molbev/msz106)
Supplement: msz106_Supplementary_Data [file msz106_supplementary_data.pdf]

Supplementary Text for
"Estimating epidemic incidence and prevalence from genomic data"

# 1 Using PMMH for phylodynamic likelihoods

As described in the manuscript, the birth-death-sampling models described in our manuscript are forward-time continuous-time Markov chains. The full epidemic trajectory $\mathcal{E}$ is assumed to be produced by this model. On the other hand, sampled phylogenies $\mathcal{T}$ can be regarded the result of *backwards-time* discrete-time Markov chains conditional on $\mathcal{E}$. Together with the unsequenced samples $\mathcal{S}$, we refer to this as the observed process $\mathcal{O}$.

The algorithm presented in the manuscript employs a bootstrap particle filter to (a) provide an estimate of the posterior probability of $\mathcal{T}, \mathcal{S}$ given a particular set of model parameters, marginalized over the unobserved trajectories $\mathcal{E}$, and to (b) sample a trajectory $\mathcal{E}$ from the probability of trajectories conditioned on the model parameters and the observed process.

Since bootstrap particle filters are usually applied exclusively to hidden Markov models (HMMs) in which the sequence of hidden states is produced by a Markov process and the sequence of observations are uncorrelated emissions from the hidden state, it is not necessarily clear that our application is appropriate. However, as we show below, the joint probability $P(\mathcal{O}, \mathcal{E})$ takes the same form as a HMM, despite the presence of backward-time correlations between the elements of the observed process $\mathcal{O}$. We demonstrate that this equivalence of form is sufficient for our application of PMMH to be correct.

## 1.1 Form of the phylodynamic likelihood

As in the manuscript, $\mathcal{O}$, the union of the sampled tree $\mathcal{T}$ and the unsequenced samples $\mathcal{S}$ (i.e. sample observations that do not correspond to

nodes on the tree) is composed of a sequence of "observations" $\mathcal{O}_j$ which include the portion of the sampled tree between observed events (coalescence events or sequenced/unsequenced sampling events) $j-1$ and $j$, including the event $j$ itself (but excluding event $j-1$).

Given that the sampled phylogeny is produced by a backward-time Markov process conditional on the trajectory (eq. 7 in the manuscript), the joint probability of the observations and the trajectory can be written

$$P(\mathcal{E},\mathcal{O}) = P(\mathcal{E}) \left[ \prod_{j=1}^{N-1} P(\mathcal{O}_j|\mathcal{O}_{j+1},\mathcal{E}) \right] P(\mathcal{O}_N|\mathcal{E}). \tag{S1}$$

Note that here we omit conditional dependence on the model parameters $\mathcal{A}_0$, $\boldsymbol{\eta}$ ,$\boldsymbol{\sigma}$ and $T$ from $P(\mathcal{E},\mathcal{O})$ and $P(\mathcal{E})$ for notational brevity.

Given that the trajectories are generated forward in time by a continuous-time Markov process, it is of course also possible to write

$$P(\mathcal{E}) = P(\mathcal{E}_1) \left[ \prod_{j=2}^{N} P(\mathcal{E}_j|\mathcal{E}_{j-1}) \right]. \tag{S2}$$

Again, for brevity we also omit the dependence on the initial conditions of the trajectory.

Combining these two equations yields

$$\begin{aligned} P(\mathcal{E},\mathcal{O}) =& P(\mathcal{E}_1)P(\mathcal{O}_1|\mathcal{O}_2,\mathcal{E}) \\ & \times \left[ \prod_{j=2}^{N-1} P(\mathcal{E}_j|\mathcal{E}_{j-1})P(\mathcal{O}_j|\mathcal{O}_{j+1},\mathcal{E}) \right] \\ & \times P(\mathcal{E}_N|\mathcal{E}_{N-1})P(\mathcal{O}_N|\mathcal{E}). \end{aligned} \tag{S3}$$

When conditioning on $\mathcal{O}_{j+1}$, only the portion of the trajectory in the interval corresponding to $\mathcal{O}_j$ affects the probability of observing the partial tree. We therefore find

$$\begin{aligned} P(\mathcal{E},\mathcal{O}) =& P(\mathcal{E}_1)P(\mathcal{O}_1|\mathcal{O}_2,\mathcal{E}_1) \\ & \times \left[ \prod_{j=2}^{N-1} P(\mathcal{E}_j|\mathcal{E}_{j-1})P(\mathcal{O}_j|\mathcal{O}_{j+1},\mathcal{E}_j) \right] \\ & \times P(\mathcal{E}_N|\mathcal{E}_{N-1})P(\mathcal{O}_N|\mathcal{E}_N). \end{aligned} \tag{S4}$$

This has the form of a HMM with autocorrelated observations. Since the observations (which make up the sampled tree we are computing the proba-

bility for) are constant during the particle filter calculation, this correlation between observations does not harm the validity of the algorithm.

This can be seen by considering that the above expression has the form

$$P(\mathcal{E}, \mathcal{O}) = P(\mathcal{E}_1) w_1(\mathcal{E}_1) \prod_{j=2}^{N} P(\mathcal{E}_j | \mathcal{E}_{j-1}) w_j(\mathcal{E}_j). \tag{S5}$$

where $w_j(\mathcal{E}_j)$ is the weight corresponding to simulated "particles" drawn from the distributions $P(\mathcal{E}_1)$ and $P(\mathcal{E}_j|\mathcal{E}_{j-1})$. This is precisely the form of the joint probability distribution targeted by the bootstrap particle filter and, by extension, PMMH. This correspondence is sufficient, since one could easily construct a sequence of *artificial* observations $y_j$ with emission probabilities defined to be $P(y_j|\mathcal{E}_j) \equiv w_j(\mathcal{E}_j)$, causing the equation to be interpreted as the joint probability for the observations and hidden state of a true HMM.

Despite this formal equivalence, there remains a subtle yet important distinction between the algorithm presented in the manuscript and the standard application of particle filters to HMMs. In contrast to the standard situation, the correlations between subsequent observations $\mathcal{O}_j$ in our application (which manifest as the dependence of the weights not only on $\mathcal{O}_j$ but also on $\mathcal{O}_{j+1}$) mean that the average of the weights computed for observation $j$ cannot be directly interpreted as the marginal probability $P(\mathcal{O}_1, \ldots, \mathcal{O}_j)$. Instead, only the *product* of these averages is meaningful and can correspond to the full marginal joint probability. While this does not impact our use of the algorithm, this acknowledgement of this fact may help the reader appreciate that information from both "past" and "future" observations are taken into account in the inference of each portion of the prevalence trajectory.

## 1.2 Correctness demonstration for large particle numbers

As further evidence that bootstrap particle filtering can indeed be applied to this system, we can easily show that the algorithm consistently estimates the marginal probability for the observations given the phylodynamic model parameters in the limit of a large number of particle simulations.

### 1.2.1 Observation 1

The first step of the filtering algorithm involves sampling $M$ independent partial trajectories $\mathcal{E}_1^{(a)} \sim P(\mathcal{E}_1)$ and defining $w_1^{(a)} = P(\mathcal{O}_1|\mathcal{O}_2, \mathcal{E}_1^{(a)})$ to be the associated weight. A new set of particles $M$ are sampled (with

replacement) from this original weighted set: $\bar{\mathcal{E}}_1^{(b)} \sim \sum_a \bar{w}_1^{(a)} \delta(\mathcal{E}_1 - \mathcal{E}_1^{(a)})$ where $\bar{w}_1^{(a)} = w_1^{(a)} / \sum_a w_1^{(a)}$.

By the law of large numbers we see that the arithmetic mean for the first set of weights approaches

$$\lim_{M\to\infty} \frac{1}{M} \sum_{a=1}^{M} w_1^{(a)} = \int P(O_1|O_2, \mathcal{E}_1) P(\mathcal{E}_1) d\mathcal{E}_1 = Z_1. \tag{S6}$$

In the same limit, the sampling distribution for $\bar{\mathcal{E}}_1^{(b)}$ becomes

$$\begin{aligned}
\lim_{M\to\infty} \sum_{a=1}^{M} \bar{w}_1^{(a)} \delta(\mathcal{E}_1 - \mathcal{E}_1^{(a)}) &= \lim_{M\to\infty} \frac{\frac{1}{M}\sum_{a=1}^{M} w_1^{(a)} \delta(\mathcal{E}_1 - \mathcal{E}_1^{(a)})}{\frac{1}{M}\sum_{a=1}^{M} w_1^{(a)}} \\
&= \frac{\lim_{M\to\infty} \frac{1}{M}\sum_{a=1}^{M} w_1^{(a)} \delta(\mathcal{E}_1 - \mathcal{E}_1^{(a)})}{\lim_{M\to\infty} \frac{1}{M}\sum_{a=1}^{M} w_1^{(a)}} \\
&= \frac{P(\mathcal{O}_1|\mathcal{O}_2, \mathcal{E}_1) P(\mathcal{E}_1)}{\int P(\mathcal{O}_1|\mathcal{O}_2, \mathcal{E}_1) P(\mathcal{E}_1) d\mathcal{E}_1} \\
&\equiv \frac{F_1(\mathcal{E}_1)}{Z_1} \equiv f_1(\mathcal{E}_1),
\end{aligned} \tag{S7}$$

where the second line holds provided the limits in the numerator and denominator exist, and that the limit in the denominator is non-zero.

## 1.3 Observation $j$

Now consider the $j$-th step, where we have $M$ partial trajectories $\mathcal{E}_j^{(a)} \sim \int P(\mathcal{E}_j|\mathcal{E}_{j-1}) f_{j-1}(\mathcal{E}_{j-1}) d\mathcal{E}_{j-1}$, where we define $f_{j-1}(\mathcal{E}_{j-1})$ to be limiting distribution of the resampled particle states for observation $j-1$. Following the same arguments above, the arithmetic mean for the $n^{\text{th}}$ set of weights approaches

$$\lim_{M\to\infty} \frac{1}{M} \sum_{a=1}^{M} w_j^{(a)} = \int P(\mathcal{O}_j|\mathcal{O}_{j+1}, \mathcal{E}_j) \int P(\mathcal{E}_j|\mathcal{E}_{j-1}) f_{j-1}(\mathcal{E}_{j-1}) d\mathcal{E}_{j-1} d\mathcal{E}_j = Z_j \tag{S8}$$

while the sampling distribution for $\bar{\mathcal{E}}_j^{(b)}$ becomes

$$\begin{aligned}
\lim_{M\to\infty} \sum_{a=1}^{M} \bar{w}_j^{(a)} \delta(\mathcal{E}_j - \mathcal{E}_j^{(a)}) &= \frac{P(\mathcal{O}_j|\mathcal{O}_{j+1}, \mathcal{E}_j) \int P(\mathcal{E}_j|\mathcal{E}_{j-1}) f_{j-1}(\mathcal{E}_{j-1}) d\mathcal{E}_{j-1}}{\int P(\mathcal{O}_j|\mathcal{O}_{j+1}, \mathcal{E}_j) \int P(\mathcal{E}_j|\mathcal{E}_{j-1}) f_{j-1}(\mathcal{E}_{j-1}) d\mathcal{E}_{j-1} d\mathcal{E}_j} \\
&\equiv \frac{F_j(\mathcal{E}_j)}{Z_j} \equiv f_j(\mathcal{E}_j)
\end{aligned} \tag{S9}$$

giving a recursion relation for $f_j(\mathcal{E}_j)$.

Using this relation, we find that

$$\begin{aligned}F_j(\mathcal{E}_j) =& P(\mathcal{O}_j|\mathcal{O}_{j+1}, \mathcal{E}_j) \int P(\mathcal{E}_j|\mathcal{E}_{j-1}) \frac{F_{j-1}(\mathcal{E}_{j-1})}{Z_{j-1}} d\mathcal{E}_{j-1} \\ =& \int d\mathcal{E}_{j-1} d\mathcal{E}_{j-2} \dots d\mathcal{E}_1 \Big[ P(\mathcal{O}_j|\mathcal{O}_{j+1}, \mathcal{E}_j) P(\mathcal{O}_{j-1}|\mathcal{O}_j, \mathcal{E}_{j-1}) \dots P(\mathcal{O}_1|\mathcal{O}_2, \mathcal{E}_1) \\ & \times P(\mathcal{E}_j|\mathcal{E}_{j-1}) P(\mathcal{E}_{j-1}|\mathcal{E}_{j-2}) \dots P(\mathcal{E}_1) \Big] \Big[ Z_{j-1} Z_{j-2} \dots Z_1 \Big]^{-1}. \quad \text{(S10)}\end{aligned}$$

### 1.3.1 Observation $N$

Finally, for the $N^{\text{th}}$ observation, the weight of a particle with state $\mathcal{E}_N^{(a)}$ is $P(\mathcal{O}_N|\mathcal{E}_N^{(a)})$, meaning that

$$\lim_{M\to\infty} \frac{1}{M} \sum_{a=1}^{M} w_N^{(a)} = \int P(\mathcal{O}_N|\mathcal{E}_N) \int P(\mathcal{E}_N|\mathcal{E}_{N-1}) f_{N-1}(\mathcal{E}_{N-1}) d\mathcal{E}_{N-1} d\mathcal{E}_N = Z_N. \quad \text{(S11)}$$

Thus, the arithmetic mean of the weights in the $N^{\text{th}}$ interval is

$$\begin{aligned}Z_N =& \int d\mathcal{E}_N d\mathcal{E}_{N-1} \dots d\mathcal{E}_1 \Big[ P(\mathcal{O}_N|\mathcal{E}_N) P(\mathcal{O}_{N-1}|\mathcal{O}_N, \mathcal{E}_{N-1}) \dots P(\mathcal{O}_1|\mathcal{O}_2, \mathcal{E}_1) \\ & \times P(\mathcal{E}_N|\mathcal{E}_{N-1}) P(\mathcal{E}_{N-1}|\mathcal{E}_{N-2}) \dots P(\mathcal{E}_1) \Big] \Big[ Z_{N-1} Z_{N-2} \dots Z_1 \Big]^{-1} \\ =& P(\mathcal{O}_N, \mathcal{O}_{N-1}, \dots, \mathcal{O}_1) \prod_{j=1}^{N-1} Z_j^{-1}. \quad \text{(S12)}\end{aligned}$$

The product of the arithmetic means of the weights in each interval therefore yields

$$\prod_{j=1}^{N} Z_1 = P(\mathcal{O}) \quad \text{(S13)}$$

which is the marginal density of the observed process (sampled phylogeny and unsequenced samples), as required.
